# Psychological Variables Related to Adaptation to the COVID-19 Lockdown in Spain

**DOI:** 10.3389/fpsyg.2020.565634

**Published:** 2020-09-16

**Authors:** Fabia Morales-Vives, Jorge-Manuel Dueñas, Andreu Vigil-Colet, Marta Camarero-Figuerola

**Affiliations:** ^1^Department of Psychology, Research Center for Behavior Assessment, Universitat Rovira i Virgili, Tarragona, Spain; ^2^Department of Pedagogy, Universitat Rovira i Virgili, Tarragona, Spain

**Keywords:** lockdown, COVID-19, coronavirus disease, psychological impact, resilience, stress

## Abstract

Recent studies show that quarantine and lockdown are effective measures for controlling COVID-19 outbreaks, but may be an unpleasant experience with psychological consequences. For this reason, the main aim of this study was to determine which personal sociodemographic and psychological variables are related to adapting to lockdown in a Spanish population. Questionnaires were administered to 2,055 individuals (60.7% women) who were resident in Spain and aged between 18 and 80 years old. We also administered some items related to feelings and behaviors during lockdown. The results showed that sex and age are variables to be taken into account. In fact, women tended to show greater stress, a more pessimistic attitude, and lower self-esteem. However, older people adapted better to lockdown although they were also more worried. Married people also adapted better although they too were more worried. The results also showed that more resilient people, with higher subjective happiness and life satisfaction, develop strategies for adapting positively to adversity, and tend to adapt better to lockdown, with more positive attitudes and behaviors. In terms of personality traits, higher neuroticism and lower extraversion were related to worse adaptation to lockdown. This study also showed that lockdown has had a negative psychological impact on those people who did not adapt well to the situation and the changes during the first 4 weeks of lockdown.

## Introduction

In the last quarter of 2019, the first cases of people who had gone down with a pneumonia of unknown origin were identified in the city of Wuhan (People’s Republic of China). The disease, caused by the severe acute respiratory syndrome–coronavirus 2 (SARS-CoV-2), was first reported to the World Health Organization (WHO) on 31 December 2019. In a relatively short period of time it spread to several countries, so on 11 March 2020 the WHO declared the pandemic derived from coronavirus disease (2019-nCoV, now renamed COVID-19). As there are no vaccines or effective treatments or preventive measures, quarantine and lockdown measures have been imposed in many countries to prevent the virus from spreading. Quarantine involves restricting the social contact of asymptomatic people who may have been exposed to a contagious disease to see whether they become ill, while lockdown involves a mass quarantine for the residents of a particular region or country to reduce spread beyond the lockdown area. Lockdown means that members of the general public are prevented from having social contact with others, and have restrictions placed on movement and traveling.

On 14 May 2020, the WHO alerted that the pandemic may impact on people’s mental health, and that depression and anxiety are increasing, especially in the most vulnerable groups (health-care workers, children, women who are juggling home-schooling or working from home and household tasks, people with pre-existing mental health conditions, etc.), although mental health services have been interrupted in many countries, and face-to-face services closed. The pandemic has involved social isolation, fear of contagion, loss of family members and even loss of income or employment for many people, all of which generates considerable distress. Although recent studies show that quarantine and lockdown are necessary because they are effective measures for controlling COVID-19 outbreaks (e.g., Lau et al., 2020; Nussbaumer-Streit et al., 2020), they can be an unpleasant experience because of the loss of freedom, the lack of contact with loved ones, boredom and uncertainty about the future and the progression of the disease, etc. The review by Brooks et al. (2020) on quarantine in previous pandemics (SARS, Ebola, and H1N1 influenza) has shown that it can have an impact on people’s psychological health, and give rise to long-lasting effects such as post-traumatic stress symptoms, confusion, and anger. Likewise, recent studies on COVID-19 have also shown the impact of quarantine and lockdown on the psychological well-being of people. For example, Moccia et al. (2020) assessed the psychological distress perceived by the general Italian population during the first phase of the COVID-19 pandemic, and the results showed that 38% of the sample suffered mild or high levels of psychological distress. Furthermore, a study carried out in the general Chinese population showed that about 53.8% of respondents suffered moderate-to-severe psychological impact in the first weeks of quarantine, 16.5% reported moderate-to-severe depressive symptoms, 28.8% reported moderate-to-severe anxiety symptoms, and 8.1% reported moderate-to-severe stress levels (Wang et al., 2020a). The longitudinal study carried out by Wang et al. (2020b) suggests that social distancing and quarantine have a greater psychological impact in the first weeks of imposition and this impact decreases after 4 weeks (Wang et al., 2020b). However, previous studies on SARS have shown that psychological problems can last for months or even years after the social distancing ends (e.g., Hawryluck et al., 2004; Liu et al., 2012).

Recent studies show that some sociodemographic variables can protect against the psychological impact of the COVID-19 quarantine and lockdown. More specifically, being a man (Brooks et al., 2020; Brouard et al., 2020), having a partner (Li et al., 2020; Moccia et al., 2020), having at least one child (Brooks et al., 2020), having confidence in the health and political system (Brouard et al., 2020), having a positive perception of public social distancing measures (Brooks et al., 2020), having daily routines (Brooks et al., 2020), and being older (Brooks et al., 2020; Brouard et al., 2020; Li et al., 2020) can lessen the impact of quarantine on psychological health. Moreover, as the study by Brooks et al. (2018) shows, highly resilient people seem to cope better with uncertainty and other problems in disaster situations (terrorism incidents, floods, etc.), so they would be expected to cope better with lockdown difficulties. Personality traits can also play a role in how lockdown is handled, because they are related to subjective wellbeing and resilience. More specifically, extraversion, neuroticism and conscientiousness are the main predictors of subjective wellbeing (e.g., Grant et al., 2009; Brajša-Žganec et al., 2011), and they are also predictors of resilience (e.g., Ercan, 2017; Oshio et al., 2018). Therefore, these traits represent personality predispositions for subjective wellbeing and resilience, so they may be relevant in predicting how lockdown is experienced. In fact, it seems that people with high extraversion levels tend to have greater difficulty in reducing social proximity and show less engagement with lockdown measures, while people with high conscientiousness levels are more engaged with these measures (Carvalho et al., 2020). However, the studies linking personality traits to subjective wellbeing and resilience suggest that extraverted people tend to experience more wellbeing and resilience, not less (e.g., Brajša-Žganec et al., 2011; Oshio et al., 2018).

Spain is one of the countries with the highest number of infections and deaths by COVID-19 worldwide. To prevent a greater spread of the virus throughout the country and to deal with the health emergency, the Spanish Government declared the state of alarm (Royal Decree 463/2020) on March 14, 2020, which led to the imposition of a national lockdown as the main measure. However, for the first 2 weeks the confinement was less severe, since exceptions were made for some services and jobs. However, in the third week (March 28, 2020) the lockdown was made stricter, and all non-essential service workers had to stay at home. Taking into account the negative effects of lockdown that have been reported in other countries, the main goal of the current study is to determine which sociodemographic and personal psychological variables are related to the ability to adapt to lockdown. More specifically, we expect to find that high levels of resilience, subjective happiness and life satisfaction, and low levels of neuroticism and extraversion, make adaptation to lockdown easier, and lead to more positive attitudes and behaviors. As previous studies show that being older can lessen the impact of quarantine on psychological health (Brooks et al., 2020; Brouard et al., 2020; Li et al., 2020), we also expect to find a relationship between age and adaptation to lockdown. Some studies have shown the importance of having a partner in this kind of situation (Li et al., 2020; Moccia et al., 2020), so we expect to find that people who are married or who have a formal partner adjust better to lockdown. We also expected to find that those people who are alone during lockdown, without a couple or other family, have greater difficulty in adapting to the situation.

We also expect that more positive attitudes and behaviors during lockdown lessen its negative psychological impact, and lead to lower levels of stress, higher levels of self-esteem and successful coping (which involves not having problems of concentrating, making decisions, playing a useful part, enjoying day-to-day activities and feeling reasonably happy) in comparison with people with negative attitudes and behaviors. As far as work is concerned, we expect that losing a job during lockdown, or being afraid of losing it, also has a psychological impact during lockdown.

Another goal of this study is to determine if there are any changes in several variables during the first 4 weeks of lockdown. We expected to find a decrease in life satisfaction, subjective happiness, self-sesteem, stress and succesful coping, but we did not exepct changes in extraversion and neuroticism, because they are personality traits, so they tend to be relatively stable.

## Materials and Methods

### Participants

The participants were 2,055 individuals (60.7% women) who were resident in Spain aged between 18 and 80 years old (*M* = 41.6, *SD* = 13.3). A total of 25.9% of the sample was single, 44.3% were married, 7.5% were divorced or separated, 12.3% lived with their partner but without being married, and 10.0% had a partner although they lived apart. Moreover, 49.6% of participants did not have children, 37.8% lived with one or more sons or daughters, and 12.6% had sons or daughters although they did not live with them. As far as work is concerned, 87.9% of the sample had a job, 6.8% had lost their job during the lockdown and 5.3% were afraid of losing it. Finally, 32.7% answered the questionnaires during the first week of lockdown, 23.4% during the second week, 25.0% during the third week, 15.7% during the fourth week and 3.2% during the fifth week.

This is not a longitudinal study, so the sample for each week consisted of different people. The mean age of the participants in the first week was 43.4 (*SD* = 14.1), 23.1% were single, 48.1% were married, 8.2% were divorced or separated, 10.7% lived with their partners but were not married, and 10.0% had a partner although they lived apart. Moreover, 46.0% did not have children, 32.9% lived with one or more of their children, and 21.1% had children but did not live with them. As far as work is concerned, 84.1% of the sample had a job, 7.5% had lost their job during the lockdown and 8.4% were afraid of losing it.

The mean age of the participants in the second week was 41.3 (*SD* = 14.9), 26.4% were single, 41.0% were married, 6.1% were divorced or separated, 14.0% lived with their partner but were not married, and 12.5% had a partner although they lived apart. Moreover, 51.1% did not have children, 35.9% lived with one or more of their children, and 13.1% had children but did not live with them. As far as work is concerned, 88.1% of the sample had a job, 7.3% had lost their job during the lockdown and 4.6% were afraid of losing it.

The mean age of the participants in the third week was 42.3 (*SD* = 11.2), 26.1% were single, 41.6% were married, 8.0% were divorced or separated, 13.8% lived with their partner but were not married, and 10.5% had a partner although they lived apart. Moreover, 53.7% of participants did not have children, 37.5% lived with one or more of their children, and 8.8% had children but did not live with them. As far as work is concerned, 89.5% of the sample had a job, 6.0% had lost their job during the lockdown and 4.5% were afraid of losing it.

The mean age of the participants in the fourth week was 40.6 (*SD* = 12.4), 25.7% were single, 48.9% were married, 8.4% were divorced or separated, 10.2% lived with their partner but were not married, 6.8% had a partner although they lived apart. Moreover, 42.41% of participants did not have children, 49.23% lived with one or more of their children, and 8.36% had children but did not live with them. As far as work is concerned, 89.4% of the sample had a job, 7.1% had lost their job during the lockdown and 3.4% were afraid of losing it. Therefore, the participants from each week have similar characteristics: most of them had a job and very few were afraid of losing it, about 40% were married, and about half of each subsample did not have any children.

Therefore, the participants from these 4 weeks have similar characteristics: most of them had a job and very few were afraid of losing it, about 40% were married, and about half of each subsample did not have any children. Since there were few subjects in the fifth week, the comparison between the different weeks does not include the participants of this week. In fact, these participants were only included for the factor analysis. The mean age of the participants in the fifth week was 40.5 (*SD* = 12.4), 41.5% were single, 32.3% were married, 3.1% were divorced or separated, 13.8% lived with their partner but were not married, 9.2% had a partner although they lived apart. Moreover, 69.2% of participants did not have children, 26.2% lived with one or more of their children, and 4.6% had children but did not live with them. As far as work is concerned, 92.3% of the sample had a job, 3.1% had lost their job during the lockdown and 4.6% were afraid of losing it.

### Measures

#### General Health Questionnaire (GHQ-12; Goldberg and Williams, 1988)

The instrument consists of 12 items (6 positive and 6 negative) that assess the severity of a mental problem over the previous few weeks. Respondents answer on a four-point Likert-type scale (from 0 to 3). Positive items are corrected from 0 (more than usual) to 3 (far less than usual) and the negative items are corrected from 3 (more than usual) to 0 (far less than usual). The study by Sánchez-López and Dresch (2008) revealed three factors in the Spanish population, which coincided with several other studies: Successful coping, Self-esteem and Stress. The factor Successful coping includes items on the difficulties of concentrating, making decisions, playing a useful part, enjoying day-to-day activities and feeling reasonably happy. The factor Self-esteem includes items about not being able to overcome difficulties, losing confidence, and thinking of oneself as worthless. The factor Stress includes items about losing sleep over worry, feeling constantly under strain, and feeling unhappy and depressed. This study shows that the questionnaire has adequate reliability and validity in the Spanish population. We found the following internal consistencies: 0.72 for Successful coping, 0.83 for Self-esteem, 0.76 for Stress, and 0.87 for the overall scores.

#### The Satisfaction With Life Scale

The Satisfaction with Life Scale questionnaire was used (SWLS; Diener et al., 1985) in the Spanish version developed by Atienza et al. (2000). This adaptation has adequate psychometric properties and an internal consistency of 0.84, the same value found in the current study. The questionnaire has a unifactorial structure made up of five items on a Likert type scale (1 = Totally disagree, 5 = Totally agree).

#### Subjective Happiness Scale (SHS; Lyubomirsky and Lepper, 1999)

This scale evaluates the degree of global subjective happiness through four items on a 7-point Likert type scale. We used the Spanish adaptation developed by Extremera and Fernández-Berrocal (2014), which has adequate internal consistency and convergent validity. In the current sample we found an internal consistency of 0.77.

#### Connor-Davidson Resilience Scale – 10 Items (CD-RISC 10; Campbell-Sills et al., 2009)

This questionnaire assesses resilience, which is understood as the development of strategies for positive adaptation to adversity. It consists of 10 Likert-type items with five response options (0 = completely disagree to 4 = completely agree). The study by Soler et al. (2016) shows that the Spanish adaptation of the 10-item version of the CD-RISC has adequate psychometric properties and a unifactorial structure. In the current sample we found an internal consistency of 0.88.

#### Big Five Inventory (BFI, John et al., 1991)

This questionnaire assesses the Big Five personality traits. We used the Spanish adaptation developed by Benet-Martínez and John (1998), although we only administered two subscales: extraversion (eight items) and neuroticism (eight items). We decided not to include the other BFI subscales so as not to further increase the time required to answer the battery of questionnaires and the descriptive items. In fact, our initial intention was to include all the subscales, but a pilot study with a few subjects revealed that they considered it too long. This could have been a problem because subjects tend not to complete the study when they consider it too long, especially when it is online and no compensation is offered for participation, as in the current study. The Spanish adaptation of this questionnaire has adequate psychometric properties, and good internal consistency and convergent validity. In the current sample we found an internal consistency of 0.84 for extraversion and 0.75 for neuroticism.

#### COVID-19 Questionnaire

We administered 15 items on adaptation to lockdown, the behaviors displayed during this situation (for example, keeping routines, using sense of humor to reduce anguish and fear, getting information only from official media, etc.), feelings about the disease (worry, fear of getting infected or that a family member may get infected, etc.) and trust in the health system and the appropriateness of lockdown. The content of these items can be seen in Table 1. All the items were answered on a 5 point Likert scale (1 = Totally disagree and 5 = Totally agree for items 1–13; 1 = Very badly and 5 = Very well for items 14 and 15). These items were written specifically for this research.

**TABLE 1:** Rotated pattern matrix of items, in bold dominant saturation.

Item	Positive	Negative
(1) I feel really worried about the COVID-19 health crisis.	−0.036	0.709
(2) I only consult official or responsible information channels for information about COVID-19.	**0.228**	0.133
(3) I am always talking about the health crisis and COVID-19.	−0.172	**0.444**
(4) I share news about COVID-19 without checking whether the information channel is official.	−0.132	**0.249**
(5) I follow certain routines (like respecting a timetable or using a particular space) when working from home or doing other daily activities.	**0.417**	0.071
(6) I follow the recommendations and prevention measures of the health authorities.	0.354	**0.369**
(7) I trust in science and in the experience of the health system.	**0.402**	0.101
(8) I take part in communal events (WhatsApp groups with neighbors, “meetings” on the balcony, volunteering, etc.).	0.171	**0.240**
(9) I use humor to reduce anguish and keep my fear in check.	**0.330**	0.039
(10) The COVID-19 quarantine is a good measure to guarantee the health of the population as a whole.	**0.417**	**0.365**
(11) I am afraid of being infected with coronavirus (COVID-19).	−0.159	**0.807**
(12) I am afraid that a loved one will be infected with coronavirus (COVID-19).	−0.113	**0.762**
(13) I think that the family atmosphere and the experience of living together during quarantine is pleasant and safe.	**0.562**	0.107
(14) As far as lockdown is concerned, how do you feel emotionally about staying so long at home?	**0.798**	−0.258
(15) To what extent have you adapted to the lockdown situation?	**0.845**	−0.256

### Procedure

The Ethical Committee of the Faculty of Education Sciences and Psychology of the Universitat Rovira i Virgili approved this project. We also obtained informed consent from all participants, in accordance with the Declaration of Helsinki. The battery of questionnaires was administered online by means of a survey designed for this purpose. The exclusion criteria were being under 18 years old, not resident in Spain, or not providing informed consent. Each questionnaire included information about the response format and the procedure for completing it. Participants had to accept the conditions of the study before participating and they could decide to drop out at any time. Confidentiality and data protection were guaranteed, and the questionnaires were completely anonymous.

We used several procedures to recruit a sample that was as heterogeneous as possible, considering the limitations imposed by the lockdown situation. Some of the participants were recruited through WhatsApp and Facebook groups during the five first weeks of lockdown in Spain, using a non-probabilistic sampling procedure known as “snowball” (Snijders, 1992). We also contacted several Spanish associations to help us disseminate the questionnaire. Several mass media published articles about this project, and included the link of the questionnaire so that their readers could answer it. Once the participants had finished the questionnaire, the website allowed them to share it with other people on the social networks (e.g., WhatsApp and Facebook). We chose the online format because the lockdown made recruitment with other procedures difficult. Several authors have suggested that psychological questionnaires can be administered online and that the results are similar to those of paper administrations (e.g., Mangunkusumo et al., 2006).

The *COVID-19 questionnaire* was specifically developed for this study. The items were written by three researchers, one of them with experience in the development of questionnaires and the other two with experience in research about life satisfaction, depressive symptomatology and social support. The content and wording of these items was also assessed by two external judges with experience in the field, who considered they were suitable for the purposes of this research and the population under study. The items are shown in Table 1.

#### Data Analysis

To assess the dimensionality of the factor structure of the *COVID-19 questionnaire*, we performed an exploratory factor analysis on the polychoric inter-item correlation matrices, using the optimal implementation of parallel analysis to determine the number of factors to retain (Timmerman and Lorenzo-Seva, 2011). The extraction method was unweighted least squares because it is more robust against the excess of skewness and kurtosis usually present in Likert-type data. Data was rotated using Promin (Lorenzo-Seva, 1999) which tends to obtain the simplest solution possible even in the presence of complex items.

The effect of sociodemographic variables on psychometric measures was analyzed using analysis of variance, or the Brown–Forsythe test when the Levene test indicated heteroscedasticity, and *post hoc* procedures (the Tuckey or Tamhane test depending on homoscedasticity).

All the data were analyzed using the program Factor (Lorenzo-Seva and Ferrando, 2013) and SPSS 25.0.

## Results

**FIGURE 1:**
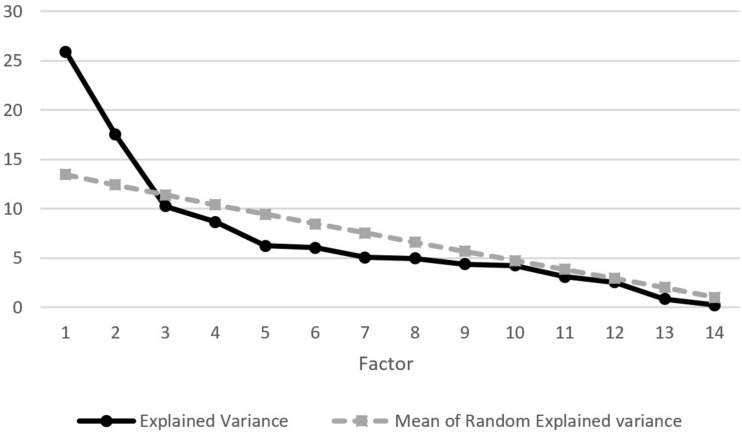
Parallel analysis of items.

We performed an exploratory factor analysis on the 15 items related to COVID-19. The value of the Kaiser–Meyer–Olkin index was 0.76, so we concluded that the correlation matrix was suitable for factor analysis. The multivariate kurtosis coefficient was 325.78 (*Z* = 70.25; *p* < 0.001). In this situation a factor analysis method that assumes normal multivariate distribution is not advisable. For this reason, we chose Unweighted Least Squares as the factor extraction method. Figure 1 shows the result of parallel analysis which advised to retain two factors. Table 1 shows the pattern matrix after oblimin rotation. As can be seen, one factor comprised positive attitudes, behaviors and feelings such as adapting well to the situation, not spreading fake news, etc., while the other one comprised negative attitudes and feelings such as being worried, having fears of being infected, etc. The correlation between both factors was *r* = 0.29 and their factor reliabilities were *r*_*θθ*_ = 0.84 and *r*_*θθ*_ = 0.83, respectively.

We computed factor scores for each individual in both factors and related them to the other variables measured. These factor scores were transformed from typical scores to T scores (i.e., mean 50 and standard deviation 10).

Table 2 shows descriptive statistics for the psychometric variables and for the extracted factors and sex effects on these variables. Women had higher levels of extraversion, neuroticism, low self-esteem and stress, and were more pessimistic about the situation and about being infected (negative factor). Most of these effects were small or, in some cases, moderate.

**TABLE 2:** Descriptive statistics and sex differences.

Variable	Mean	*SD*	Men	Women	*p*	Cohen’s *d*
Life satisfaction	18.23	3.68	18.08	18.28	n.s	
Resilience	26.89	6.13	27.45	26.65	<0.05	0.03
Happiness	20.63	4.54	20.40	20.76	n.s.	
Extraversion	27.22	6.42	25.94	27.65	<0.01	0.27
Neuroticism	23.93	4.76	22.77	24.25	<0.01	0.52
Successful coping	10.00	2.77	10.15	9.97	n.s.	
Self-esteem	8.80	2.79	9.17	8.68	<0.01	0.18
Stress	3.97	2.37	3.47	4.16	<0.01	0.29
Positive	50.00	10.00	49.30	50.09	n.s.	
Negative	50.00	10.00	47.03	50.43	<0.01	0.34

Table 3 shows product moment correlations between all psychometric measures and age. As can be seen, the positive factor was more related to all variables than the negative factor. In this regard, the people that best adapted to the lockdown situation showed higher levels of life satisfaction, resilience, happiness, extraversion, self-esteem and successful coping, and lower levels of neuroticism and stress with correlation coefficients ranging from *r* = 0.170 to *r* = 0.472 in absolute values. On the other hand, the people who worried most and were more afraid of being infected showed more stress and neuroticism, low successful coping and low self-esteem, with correlation coefficients ranging from *r* = 0.072 to *r* = 0.275 in absolute values. Age was related to both factors, positive *r* = 0.224 and negative *r* = 0.166, showing that older people tend to adapt better to lockdown but are also more worried.

**TABLE 3:** Product moment correlation between measures.

				Life					Succ.		
	Positive	Negative	Age	satisf.	Resilience	Happiness	Extrav.	Neurot.	coping	Self-esteem	Stress
Positive	–										
Negative	0.29**	–									
Age	0.23**	0.17**	–								
Life satisfaction	0.39**	−0.07**	0.16**	–							
Resilience	0.36**	0.01	0.12**	0.44**	–						
Happiness	0.36**	0.01	0.12**	0.57**	0.55**	–					
Extraversion	0.17**	−0.07**	0.07**	0.28**	0.44**	0.40**	–				
Neuroticism	−0.27**	0.16**	−0.17**	−0.37**	−0.52**	−0.54**	−0.23**	–			
Successful coping	0.42**	−0.14**	0.16**	0.27**	0.27**	0.33**	0.11**	−0.29**	–		
Self-esteem	0.47**	−0.15**	0.17**	0.39**	0.41**	0.50**	0.21**	−0.47**	−0.66**	–	
Stress	−0.43**	0.28**	−0.13**	−0.28**	−0.27**	−0.34**	−0.08**	0.40**	0.60**	0.77**	–

**p < 0.01.

Table 4 shows the effects of the number of weeks of lockdown on psychometric measures. As can be seen, the weeks locked down reduced life satisfaction, happiness, successful coping and self-esteem, and increased stress. All these effects were small and *post hoc* procedures showed that in all cases the significant differences were between the first and third week of lockdown. In the case of self-esteem and stress the difference between the first and fourth week was also significant, but no other difference was significant, which seems to show that there was an increase in stress levels related to the change from partial lockdown to total lockdown and a subsequent stabilization.

**TABLE 4:** Analyses of variance of the effect of week on measures.

	Week					
	
	1	2	3	4	*p*	η^2^
Life satisfaction	18.74	18.06	17.90	18.12	<0.01*	0.01
Resilience	27.50	26.75	26.40	26.73	n.s.	
Happiness	21.22	20.31	20.40	20.87	<0.05	0.007
Extraversion	27.87	26.95	26.88	27.23	n.s.	
Neuroticism	23.64	23.88	24.18	23.74	n.s.	
Successful coping	10.19	10.05	9.67	10.18	<0.01*	0.01
Self-esteem	9.20	8.88	8.46	8.59	<0.01*	0.015
Stress	3.56	3.72	4.30	4.39	<0.01*	0.023
Positive	50.72	49.69	49.38	49.74	n.s.	
Negative	50.17	49.55	49.28	49.48	n.s.	

*The Brown–Forsythe Statistic was used due to lack of homoscedasticity.

Table 5 shows the effect of job status on the measures. Job status refers to having lost one’s job or not because of the COVID-19 crisis and having a job but being afraid of losing it because of COVID-19. As can be seen, this variable had only small effects on life satisfaction, successful coping, self-esteem and stress. *Post hoc* procedures showed that all these effects were due to the difference between people who had not lost their jobs and people who still had a job but were afraid of losing it. This last group showed lower life satisfaction, lower successful coping, lower self-esteem and higher levels of stress.

**TABLE 5:** Analysis of variance of the effect of losing a job because of COVID-19 on measures (only significant effects are shown).

			No, but I’m		
	No	Yes	afraid I will	*p*	η^2^
Life satisfaction	18.35	17.52	17.21	<0.01	0.007
Successful coping	10.10	9.83	8.93	<0.01*	0.012
Self-esteem	8.88	8.25	7.85	<0.01*	0.01
Stress	3.93	4.32	4.68	<0.05*	0.006

*The Brown–Forsythe Statistic was used due to lack of homoscedasticity.

Table 6 shows the effects of civil status on positive and negative factors. As can be seen, both measures were sensitive to this variable and effect sizes were low. *Post hoc* procedures showed that married people scored higher than other groups on the positive factor and people with a partner but not living together showed the lowest levels of adaptation to lockdown. For the negative factor, married people showed higher levels of worry than the other groups. Finally, being locked down with your partner or with your family showed a small increase in the positive factor.

**TABLE 6:** Analyses of variance of the effect of civil status and lockdown partners on measures.

	Civil status					
	Single	Partner but living apart	Married/partner	Divorced	*p*	η^2^
Positive	48.51	45.54	51.41	49.94	<0.01*	0.037
Negative	47.38	47.15	51.11	49.75	<0.01	0.033

	**Who is with you during lockdown**					
	
	**Nobody**	**Couple**	**Couple and sons**			

Positive	47.81	51.03	50.02		<0.01	0.008
Negative	48.8	50.8	50.10		n.s.	

*The Brown–Forsythe Statistic was used due to lack of homoscedasticity.

## Discussion

Several studies show that quarantine and lockdown measures have a negative impact on the population (e.g., Moccia et al., 2020; Wang et al., 2020a). For this reason, the main goal of the current study was to determine which sociodemographic and psychological variables are related to adaptation to lockdown. This information may be useful for detecting which people are especially vulnerable in this situation. According to the results, there are several important variables, but the fact that for many of them the effect sizes were small suggests that, in general, the lockdown had little impact on the general Spanish population, which is a positive result. Therefore, it seems that the sample adapted quite well to the constraints of staying at home.

The results show that sex and age are variables to be taken into account. In fact, women tend to show a more pessimistic attitude in this situation: they worry more about the health crisis, are more afraid of getting infected or relatives getting infected, and spend more time talking about the disease. Women also show more stress and less self-esteem than the usual, which means that the psychological impact is worse for them than for men. This is congruent with the review by Brooks et al. (2020) on previous pandemics, which shows that being a man is one of the sociodemographic variables that can act as a protective factor. Older people also adapted better to lockdown, although they were also more worried. The review by Brooks et al. (2020) on previous pandemics and some recent studies on COVID-19 also show that being older is a protective variable. Therefore, it seems that younger people have more problems adapting to lockdown. In terms of civil status, as expected, married people showed better adaptation, although they were also more worried, which is understandable given that they may be afraid that the pandemic will affect their partner, children, etc. In contrast, people with a romantic relationship, but not living together, showed a worse adaptation than the other groups. The results also show that those people locked down with the couple or the family adjusted better than people alone, as expected. These results are congruent with previous studies that show the importance of having a partner in this kind of situation (Li et al., 2020; Moccia et al., 2020).

As far as the relationship between psychological variables and adaptation to lockdown is concerned, the results show that more resilient people develop strategies to positively adapt to adversity, tend to adapt better to lockdown, and have more positive attitudes and behaviors. This means that they tend to establish routines during lockdown; for example, they separate teleworking times and places from leisure times and places, they tend to use sense of humor to reduce anguish and fear, they believe in the importance of lockdown, and they trust in the science and health system. This result was expected because the study by Brooks et al. (2018) shows that highly resilient people seem to cope better with uncertainty and other problems in disaster situations such as terrorist incidents or floods. Likewise, the current study also shows that more optimistic and positive people, with greater subjective happiness and life satisfaction, tend to adapt better to lockdown, and have more positive attitudes and behaviors. Therefore, according to these results, people who are more resilient, happier and with higher life satisfaction (which is the cognitive component of subjective wellbeing) tend to adapt to lockdown better. All these variables are related to personality traits (e.g., Soto, 2015; Suldo et al., 2015; Ercan, 2017). In fact, traits such as extraversion or neuroticism represent personality predispositions to resilience and subjective wellbeing (e.g., Grant et al., 2009; Brajša-Žganec et al., 2011; Oshio et al., 2018), which may explain the relationship between these traits and the adaptation to lockdown. In the current study, as expected, higher neuroticism was related to worse adaptation to lockdown and higher levels of worry and fear about COVID-19. In fact, many studies show that higher levels of neuroticism are related to more stress and worse coping in several events (e.g., Gallagher, 1990; Gunthert et al., 1999). However, we did not find the expected relationship between extraversion and adaptation to lockdown. More specifically, we expected to find a negative correlation between extraversion and adaptation to lockdown, because extraverted people have more need of social contact, so we expected that being locked down at home could be a more negative experience for them. In fact, the study by Carvalho et al. (2020) shows that people with high extraversion levels tend to find it more difficult to reduce social proximity, and show less engagement with lockdown measures. But the results of the current study suggest just the opposite, as we found a positive correlation between the two variables, which means that higher extraversion is related to better adaptation. One possible explanation of this result is the social characteristics of the Spanish population. In Spain the social networks are strong, which can make it easy to maintain social contact even in a confined situation with neighbors or online with tools such as Facebook, Instagram, etc. In fact, during lockdown many Spanish people have increased contact with neighbors from their balconies. Moreover, communal events have been organized throughout the lockdown, such as the daily applause for the health personnel. Other examples are bingo games or musical events. This situation may have mitigated the negative effect of confinement on the more extraverted people in Spain. Another possible explanation is the positive relationship that extraversion has with other variables such as resilience or subjective wellbeing (e.g., Brajša-Žganec et al., 2011; Oshio et al., 2018). In fact, in the current study a positive relationship has also been found between extraversion and resilience. The fact that extraverted people tend to be more resilient, and are more predisposed to experience subjective wellbeing, may help them to better resist the difficulties associated with lockdown. To sum up, the results of the current study suggest that more extraverted people with lower neuroticism tend to adapt better to lockdown.

With regard to the psychological impact of the lockdown, worse adaptation to the situation is related to lower levels of successful coping than usual. This involves greater difficulty in concentrating, making decisions, playing a useful part or enjoying day-to-day activities. It is also related to lower levels of self-esteem than usual, with feelings of not being able to overcome difficulties, losing confidence, and thinking of oneself as worthless. Furthermore, it is related to higher levels of stress than usual, which means losing sleep over worry, feeling constantly under strain and feeling unhappy and depressed. Recent studies in other countries have also shown the negative effects of lockdown. For example Moccia et al. (2020) in Italy and Wang et al. (2020a) in China have revealed that many people suffered from psychological distress, with anxiety and depressive symptoms.

Another variable that had a pyschological impact during lockdown was job status, although effects were only small. More specifically, people who had a job but were afraid of losing it had lower levels of life satisfaction, successful coping and self-esteem, and higher levels of stress than people who were not afraid of losing their jobs. Therefore, this fear of losing their job increased anxiety and reduced concentration, the ability to make decisions and the enjoyment of daily life activities.

In the first 4 weeks of lockdown, there was a small but significant decrease in life satisfaction and happiness between the first and the third week. Moreover, the levels of stress increased between the first and the third week, and successful coping decreased during the same period, which means that people found it more difficult to concentrate, make decisions, play a useful part or enjoy day-to-day activities during the third week. However, in the fourth week these variables stabilized. This negative change in the third week may be explained by the fact that the first 2 weeks of the lockdown in Spain was only partial. In the third week stricter restrictions were imposed, which seems to have generated greater psychological distress in the sample. Therefore, it seems that the transition from a partial to a full lockdown, not only the length of the lockdown, is a factor that also has psychological impact. Although in the current study there was a stabilization in the fourth week, Wang et al. (2020b) found that this stabilization occurred later, after the fourth week. To sum up, the current study shows several sociodemographic and psychological variables that may affect how people adapt to a situation as stressful as confinement. This information may be useful for similar situations in the future so that strategies can be developed to rapidly detect the most vulnerable people and provide them with psychological advice and support. Our results indicate that this advice and support should promote more positive behaviors during a lockdown, such as establishing routines (separating teleworking times and places from leisure times and places), or getting information only from official media. It should also aim to increase confidence in the health system and belief in the lockdown by providing objective data to correct false perceptions and hoaxes. Likewise, it should provide strategies to better cope with stress, anxiety and uncertainty by promoting psychological well-being and preventing loss of self-esteem.

A limitation of the current study is that only two of the Big Five personality traits have been assessed: extraversion and emotional stability. Further studies should be made that include the other traits, especially conscientiousness, because they may also play an important role. Moreover, the results suggest that lockdown did not have a great impact on the general Spanish population, but other studies should focus on vulnerable groups (for example, people with mental disorders or people under great pressure during the lockdown, such as health professionals). Further studies should also be done with longitudinal data so that we can better understand the different phases that people experience during lockdown. Although the current study provides important information about this, it should be taken into account that the sample for each week consists of different people, which is a limitation. However, the current study provides valuable information not only about adaptation to lockdown but also about the psychological impact of lockdown on the Spanish population. It would be interesting to carry out a further study with the same variables in order to determine the changes undergone by the time lockdown finishes, and a third study some months later when the situation has returned to normal.

## Data Availability Statement

The raw data supporting the conclusions of this article will be made available by the authors, without undue reservation, to any qualified researcher.

## Ethics Statement

The studies involving human participants were reviewed and approved by The Ethical Committee of the Faculty of Education Sciences and Psychology of the Universitat Rovira i Virgili approved this project. The patients/participants provided their written informed consent to participate in this study.

## Author Contributions

FM-V contributed to the design of the study, carried out part of the statistical analyses, supervised the research, wrote most of the article, and provided the final approval of the version to be published. J-MD formulated the research question, supervised the research, contributed to disseminate the questionnaires, and wrote part of the article. AV-C was responsible for the statistical design of the study, wrote part of the article, and provided the final approval of the version to be published. MC-F contacted the associations, contributed to disseminate the questionnaires, and wrote part of the article. All authors contributed to the article and approved the submitted version.

## Conflict of Interest

The authors declare that the research was conducted in the absence of any commercial or financial relationships that could be construed as a potential conflict of interest.
